# Alendronate Functionalized Mesoporous Bioactive Glass Nanospheres

**DOI:** 10.3390/ma9030135

**Published:** 2016-02-26

**Authors:** Elisa Boanini, Silvia Panseri, Fabiola Arroyo, Monica Montesi, Katia Rubini, Anna Tampieri, Cristian Covarrubias, Adriana Bigi

**Affiliations:** 1 Department of Chemistry “G. Ciamician”, University of Bologna, Via Selmi 2, Bologna 40126, Italy; katia.rubini@unibo.it (K.R.); adriana.bigi@unibo.it (A.B.); 2 Institute of Science and Technology for Ceramics, National Research Council of Italy, Via Granarolo 64, Faenza 48018, Italy; silvia.panseri@istec.cnr.it (S.P.); monica.montesi@istec.cnr.it (M.M.); anna.tampieri@istec.cnr.it (A.T.); 3 Laboratory of Nanobiomaterials, Institute for Research in Dental Sciences, Faculty of Dentistry, University of Chile, Santiago 8380492, Chile; fabiola.arroyo@gmail.com (F.A.); ccovarrubias@odontologia.uchile.cl (C.C.)

**Keywords:** mesoporous, bioactive glass nanospheres, alendronate, osteoclast inhibition, anti-cancer

## Abstract

In this work we synthesized mesoporous bioactive glass nanospheres (nMBG) with the aim to utilize them as substrates for loading one of the most potent amino-bisphosphonates, alendronate (AL). The results of the chemical and structural characterization show that the nMBG display a relatively high surface area (528 m^2^/g) and a mean pore volume of 0.63 cm^3^/g, both of which decrease on increasing alendronate content. It is possible to modulate the amount of AL loaded into the nanospheres up to a maximum value of about 17 wt %. *In vitro* tests were performed using a human osteosarcoma cell line (MG63) and a murine monocyte/macrophage cell line as osteoclast model (RAW 264.7). The results indicate that even the lower concentration of alendronate provokes decreased tumor cell viability, and that osteoclast activity exhibits an alendronate dose-dependent inhibition. The data suggest that nMBG can act as a suitable support for the local delivery of alendronate, and that the antiresorptive and antitumor properties of the functionalized mesoporous nanospheres can be modulated by varying the amount of alendronate loading.

## 1. Introduction

Osteosarcoma (OS) is the most common primary malignant bone tumor in children and adolescents, with a second peak in incidence in adults over the age of 50 [1]. The mainstay of curative osteosarcoma treatment is surgery (often amputation). However, the survival of patients with OS treated with surgery alone is less than 20% [2]. The basis for the aggressiveness of this tumor is largely unknown. However, although little is known on the mechanisms by which OS destroys the hard matrix of the skeleton, the role of osteoclasts, primary cells involved in bone matrix solubilization, seems to be fundamental in the cancer progression. In detail, recent findings have shown that the induction of osteoclast activity by tumor cells is particularly increased in those patients with aggressive OS [3,4]. Recent studies in *in vivo* models indicate that bisphosphonates (BPs) can inhibit the tumor local expansion and the formation of metastases [5]. In fact, BPs, which are extensively employed for the management of specific disorders of bone metabolism characterized by abnormally increased bone mass resorption, display also antitumor and anti-angiogenetic properties [6,7,8]. BPs derive chemically from pyrophosphates and are characterized by a backbone structure of P-C-P where P is a phosphonate group. Individual BPs differ in the two covalently bound side chains, R_1_ and R_2_, which complete the tetra valence of the carbon atom. The affinity for the mineral phase of bone is enhanced when R_1_ is an hydroxyl group, whereas the presence of a nitrogen atom in the R_2_ side chain positively influences the anti-resorptive potency of BPs. Amino BPs (N-BPs), such as alendronate (AL), inhibit farnesyl pyrophosphate (FPP) synthase, a major enzyme in the mevalonate pathway, and, as a consequence, hinder most osteoclast activities [9]. Although clinical studies have reported successful results of long-term uses of N-BPs [10], a vast number of adverse side effects, including osteonecrosis of the jaw and increased risk of subtrochanteric fractures, have been reported [11,12,13]. These drawbacks have stimulated the development of strategies for alternative administration of BPs, such as local release at specific bone sites [14]. Most of these studies have been performed using calcium phosphates as substrates onto which BPs are loaded through chemisorption from solution or incorporated through direct synthesis [15,16,17,18,19,20,21,22,23]. Moreover, a few studies have explored the possibility of using siliceous ordered mesoporous materials for controlled delivery of BPs [24,25].

Pure silica materials such as MCM-41 and SBA-15 with ordered mesoporous structure and high surface area have been also studied as matrices for alendronate adsorption and release [24]. Ordered mesoporous materials with CaO–SiO_2_–P_2_O_5_ bioactive glass (BG) composition can be also prepared [26]. BG is an osteoconductive and osteostimulative material able to bond closely with the host bone tissue through the formation of an apatite layer. In addition BG stimulates osteoblast proliferation and differentiation by enhancing the expression of potent osteoblast mitogenic growth factors [27]. Microsized BG particles with SBA-15 type structure (MBG) can be produced by the sol-gel technique. More recently, the synthesis of MBG nanospheres (nMBG) with particle size in the 50–100 nm range, having high surface area and mesoporous volume has been also reported [28]. The incorporation of AL into nMBG could produce a system that couples AL delivery with the properties of the nanodimensional BG material.

In this study we prepared nano-sized bioactive glass nanospheres (nMBG) loaded with increasing amounts of alendronate. The combined capability of the developed drug system as inhibitor on cancer cells viability and as inhibitor of bone resorption was investigated *in vitro* through determination of tumor cell proliferation/survival, and osteoclast activity.

## 2. Materials and Methods

### 2.1. Preparation and Characterization of Mesoporous Bioactive Glass Nanospheres

nMBG were synthesized by a hydrothermal method [28] using cetyltrimethylammonium bromide (CTAB) and poly(vinylpyrrolidone) (PVP) as co-templates. In a typical experiment, 1 g PVP and 0.46 g NaOH were first dissolved in 120 mL distilled water. Then, 1.4 g CTAB was added to the PVP-NaOH solution and stirred for 1 h. Then, tetrahydrate calcium nitrate (Ca(NO_3_)_2_·4H_2_O), tetraethyl orthosilicate (TEOS) and triethyl phosphate (TEP) were added with vigorous stirring. The molar ratio of Ca:P:Si was 15:5:80. After stirring for 24 h, the milk-like mixture was sealed in Teflon-lined autoclaves at 80 °C for 48 h. The products were collected by centrifugation and washed by water and ethanol, 3 times with each. Then the collected powders were dried at 80 °C overnight and calcined at 550 °C for 5 h to remove any remaining PVP and CTAB.

Loading of alendronate on nMBG was carried out in MilliQ water at different concentrations of sodium alendronate trihydrate (C_4_H_12_NaNO_7_P_2_·3H_2_O: 0.25, 0.5, 1 mg/mL). Samples were labelled as AL25, AL50 and AL100, respectively. The reaction was performed on 500 mg nMBG/100 mL solution at 37 °C, under stirring for 24 h. Then, the products were centrifuged at 10,000 rpm for 30 min, repeatedly washed with double distilled water and dried at 37 °C.

Alendronate content was determined spectrophotometrically via complex formation with Fe(III) ions [29] in the aqueous solution isolated after centrifugation. A Varian Cary50Bio instrument (λ = 290 nm) was used.

Attenuated total reflectance with Fourier transform infrared spectroscopy (ATR-FTIR) was carried out on an Agilent Cary 630 ATR–FTIR spectrometer (Agilent, Santa Clara, CA, USA).

Thermogravimetric analysis was carried out using a Perkin–Elmer TGA-7 (Perkin Elmer, Monza, Italy). Heating was performed in a platinum crucible in air flow (20 cm^3^/min) at a rate of 10 °C/min up to 800 °C. The samples weights were in the range 5–10 mg.

For transmission electron microscopy (TEM) investigations, dry samples were suspended in ethanol after sonication, and then were transferred onto holey carbon foils supported on conventional copper microgrids. A Philips CM100 transmission electron microscope (Philips, Leiden, The Netherlands), operating at 80 kV was used.

X-ray diffraction (XRD) analysis was carried out by means of a PANalytical X’Pert PRO powder diffractometer (PANalytical, Almelo, The Netherlands) equipped with a fast X’Celerator detector. CuKα radiation was used (40 mA, 40 kV). For phase identification the 2θ range was investigated from 10 to 60, 2θ degrees with a step size of 0.1° and time/step of 100 s.

Textural characterization of materials was carried out by N_2_ adsorption at 77 K in a Micromeritics ASAP 2010 sorptometer (Micrometrics, Norcross, Georgia). The specific surface areas (SSA) were obtained using the Brunauer-Emmett-Teller (BET) method. Pore diameter was estimated from the pore size distribution curves obtained by the Barrett-Joyner-Halenda model.

### 2.2. Preparation and Characterization of Disk-Shaped Samples

*In vitro* tests were performed on disk-shaped samples (∅ = 6.0 mm). Each disk was prepared by pressing 30 mg of powder into cylindrical molds by using a standard evacuable pellet die (Hellma, Mühlheim, Germany), and sterilized using gamma rays (Cobalt-60) at a dose of 25 kGy.

Static contact angle measurements were performed on disk-shaped samples. A KSV CAM101 instrument (Nordtest srl, GI, Serravalle Scrivia, Italy) was used under ambient conditions for recording the side profiles of deionized water drops for image analysis. The shape of the drop was recorded in a time range of 0–30 s, by collecting an image every 0.033 s. At least three drops were observed for each sample.

For atomic force microscopy (AFM) imaging a Veeco Nanoscope 3D instrument (Veeco, Oyster Bay, NY, USA) was used. The samples were analyzed in tapping mode using an E scanner (maximum scan size 15 μm) and phosphorus (n) doped silicon probes (spring constant 20–80 N/m; resonance frequency 250–290 kHz; nominal tip radius < 10 nm). Roughness parameters, namely arithmetic mean roughness (*R*_a_), root-square roughness (*R*_q_), and the vertical distance between the highest and lowest points within the evaluation length (*R*_t_), were recorded.

Mineralization tests on disk-shaped samples were performed in modified simulated body fluid solution (1.5 SBF). The modified SBF was prepared by dissolving reagent grade NaCl, KCl, NaHCO_3_, KHCO_3_, Na_2_HPO_4_·12H_2_O, MgSO_4_·7H_2_O, MgCl_2_·6H_2_O, and CaCl_2_·12H_2_O into double-distilled water and buffering at pH 7.4 with Hepes (2-[4-(2-hydroxyethyl)piperazin-1-yl]ethanesulfonic acid) and NaOH [30] Each disk-shaped sample was incubated in 25 mL of 1.5 SBF solution at 37 °C for up to 7 days. Afterwards, samples were abundantly rinsed with distilled water and dried at 37 °C.

Morphological investigations of the samples before and after exposure to SBF solution were performed using a Philips XL-20 scanning electron microscope (Philips) operating at 15 kV. The samples were sputter coated with gold before examination. Energy dispersive X-ray analysis (EDX, Philips) analyses were carried out on uncoated specimens.

### 2.3. In Vitro Study

#### 2.3.1. Cell Cultures

Human Osteosarcoma cell line, MG63, purchased from Lonza (Basel, Switzerland) were cultured in Dulbecco modified Eagle’s medium (DMEM)/F12 Medium (Gibco, Billings, MT, USA), containing 1% penicillin-streptomycin (100 U/mL-100 µg/mL) supplemented with 10% fetal bovine serum (FBS) and kept at 37 °C in an atmosphere of 5% CO_2_. Samples were placed one per well in a 24-well plate and a drop of 20 µL containing 1.00 × 10^3^ cells (2.5 × 10^3^ cells/cm^2^) was seeded on the center of the upper sample surface allowing cell attachment for 30 min in the incubator, before addition into each well of 1.0 mL of cell culture medium.

Murine monocyte/macrophage cell line RAW 264.7, obtained from ATCC cell bank (Manassas, VA, USA), was used as model of osteoclastogenesis [31,32]. RAW 264.7 cells were cultured in DMEM high glucose (Gibco), 10% FBS and 1% penicillin-streptomycin (100 U/mL-100 µg/mL). RAW 264.7 cells at a concentration of 1.5 × 10^3^ cells/cm^2^ were seeded in 0.4 µm pore size 24-well inserts (Merck Millipore, Darmstadt, Germany), and the samples were placed one per well in the 24-well plate. To initiate osteoclasts (OCLs) differentiation, 25 ng/mL soluble Receptor Activator for Nuclear Factor kB Ligand (sRANKL, Sigma-Aldrich, St Louis, MO, USA) was added to the culture.

All cell-handling procedures were performed in a sterile laminar flow hood. All cell-culture incubation steps were performed at 37 °C with 5% CO_2_.

#### 2.3.2. Cell Viability Assay

MTT reagent (3-(4,5-dimethylthiazol-2-yl)-2,5-diphenyltetrazolium bromide) (Invitrogen, Carlsbad, CA, USA) was prepared at 5 mg/mL in 1× PBS. Samples seeded with MG63 cells were incubated with the MTT reagent 1:10 for 2 h at 37 °C. Medium was discarded and cells incubated with 200 µL of dimethyl sulfoxide for 15 min. In this assay, the metabolically active cells react with the tetrazolium salt in the MTT reagent to produce a formazan dye that can be observed at λ_max_ = 570 nm, using a Multiskan FC Microplate Photometer (Thermo Fisher Scientific Inc., Waltham, MA, USA) [33]. This absorbance is directly proportional to the number of metabolically active cells. Mean values of absorbance were determined. Three samples per time point per group (day 1, 3, and 7) were analyzed.

#### 2.3.3. Actin Filament Staining

Actin filament staining was performed to assess osteoclastogenesis. In detail RAW 264.7 cells, grown in 24-well inserts, of nMBG group were washed with phosphate buffered saline (PBS 1×) for 5 min, fixed with 4% (*w*/*v*) paraformaldehyde for 15 min and washed with PBS 1× for 5 min. Permeabilization was performed with 1× PBS with 0.1% (*v*/*v*) Triton X-100 for 5 min. Fluorescein isothiocyanate (FITC) conjugated Phalloidin (Invitrogen) 38 nM in 1× PBS was added for 20 min at room temperature in the dark [34]. Cells were washed with 1× PBS for 5 min and incubated with nuclear stain 4',6-diamidino-2-phenylindole (DAPI) (300 nM, Invitrogen) in 1× PBS for 5 min.

At each time point, one sample per group with MG63 seeded was fixed with 4% (*w*/*v*) paraformaldehyde for 15 min, and cell nuclei were stained with DAPI. Images were acquired by an Inverted Ti-E fluorescence microscope (Nikon Corporation, Tokyo, Japan).

#### 2.3.4. TRAP Activity Evaluation

Tartrate resistant acid phosphatase (TRAP), highly expressed by osteoclasts, was measured according to established protocol [35]. Briefly cells, grown in 24-well inserts, were lysed in 1 M NaCl with 0.2% Triton X-100. Lysate was incubated with 50 mL of 5 mM *p*-nitrophenyl phosphate (Sigma-Aldrich, St. Louis, MO, USA) in 25 mM Na-acetate/20 mM Natartrate, pH 4.8 at 37 °C for 30 min. The reaction was stopped by adding 0.5 M NaOH. Then 100 μL of the resulting supernatant was transferred into a 96-well plate, and read by a plate reader at 405 nm. Three samples per time point per group (day 3 and 7) were analyzed. The data were reported as the percentage of the enzymatic activity with respect to the nMBG group.

#### 2.3.5. Quantitative Real-Time Polymerase Chain Reaction (qPCR)

At day 7, RAW 264.7 cells grown in 0.4 µm pore size 24-well inserts with the samples placed one per well in the 24-well plate were lysed and total RNA extraction was performed by use of Tri Reagent, followed by the Purelink RNA Mini kit according to the manufacturer's instructions. nMBG group was used as control. RNA integrity was analyzed by native agarose gel electrophoresis and quantification performed by the Qubit^®^ 2.0 Fluorometer (Invitrogen) together with the Qubit^®^ RNA BR assay kit, following the manufacturer’s instructions. Total RNA (500 ng) was reverse transcribed to cDNA using the High-Capacity cDNA Reverse Transcription Kit, according to the manufacturer’s instructions. Quantification of gene expression for Catepsin K (CtsK, Mm00484039), Osteoclast-associated immunoglobulin-like receptor (Oscar, Mm00558665), and the housekeeping gene glyceraldehyde 3-phosphate dehydrogenase (GAPDH, Mm99999915) (Life Technologies, Carlsbad, CA, USA) was performed with the StepOne™ Real-Time PCR System (Applied Biosystems, Foster City, CA, USA). Experiment was done in triplicate, using three technical replicates for each experiment. Data was collected using the OneStep Software (version 2.2.2, Applied Biosystems) and relative quantification was performed using the comparative threshold (*C*_t_) method (ΔΔ*C*_t_), where relative gene expression level equals 2^−ΔΔ*C*^_t_ [36].

#### 2.3.6. Statistical Analysis

Results were expressed as Mean ± SEM (standard error mean) plotted on a graph. MTT results analysis was made by two-way analysis of variance, followed by Bonferroni’s *post-hoc* test. Gene expression profiling was analyzed by one-way ANOVA, followed by “Tukey’s Multiple Comparison Test”. Statistical analysis was performed by the GraphPad Prism software (version 5.0, La Jolla, CA, USA), with statistical significance set at *p* ≤ 0.05.

## 3. Results and Discussion

### 3.1. Chemical and Structural Characterization

**Figure 1:**
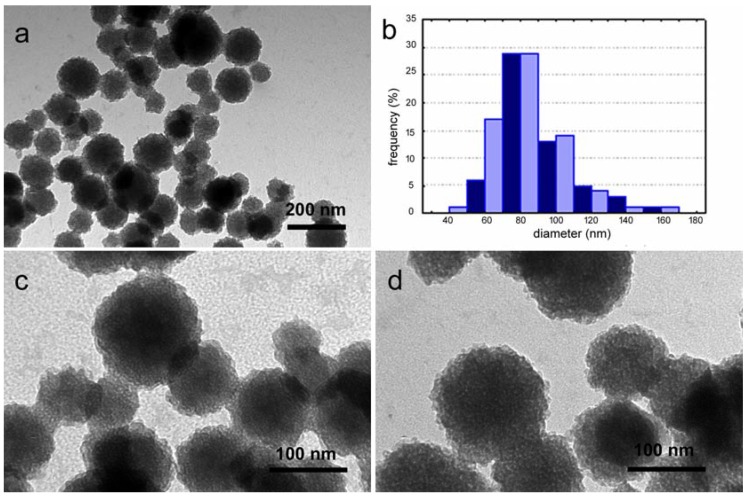
Transmission electron microscopy (TEM) images of as-prepared mesoporous bioactive glass nanospheres (nMBG) (**a**,**c**) with relative size distribution (**b**); and of AL100 (**d**).

The as-prepared nanospheres (nMBG) exhibit a spherical morphology (Figure 1a) and a size distribution between 50 and 150 nm (Figure 1b). The high magnification TEM image reported in Figure 1c reveals the internal mesoporous structure of the nanospheres. In agreement, the results of porosity investigation indicate that the nMBG display a relatively high surface area (528 m^2^/g) and a mean pore volume of 0.63 cm^3^/g (Table 1).

nMBG nanospheres display a very high loading efficiency of alendronate. Loading was performed through immersion in solutions at different AL concentration up to 1 mg/mL. The results of the spectrophotometric analysis of the AL chromophoric complex with FeIII ions [29] indicate that the amount of AL loaded into the nanospheres increases as the bisphosphonate concentration increases up to about 17 wt % (Table 1). This value represents the maximum amount of AL that can be adsorbed into the nanoparticles, since immersion in solutions at greater concentration than 1 mg/mL did not yield greater incorporation. Comparison with the amount of AL present in solution indicates that the efficiency of AL loading is about 100%, independent of concentration. Total calcium content in the nanospheres is about 20 wt % determined through energy dispersive X-ray analysis (EDX). No significant variation can be appreciated after AL loading.

**Figure 2:**
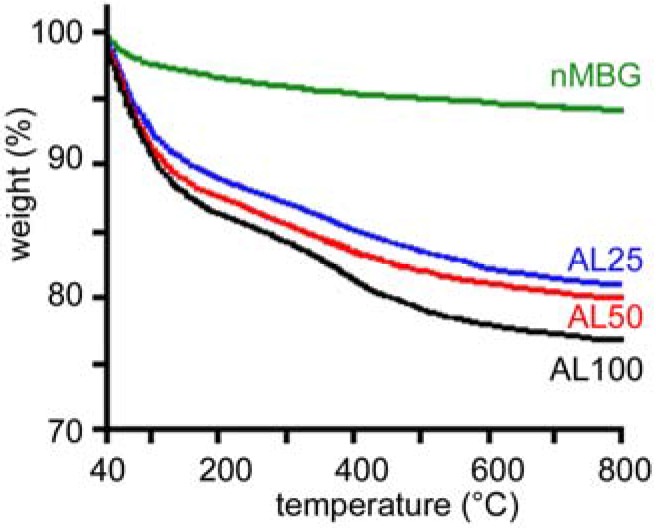
Thermogravimetric plots of the samples with different alendronate content.

The increase of the amount of AL incorporated into the nanospheres can be followed also by thermogravimetric analysis. The comparison between the thermogram of nMBG and those of AL loaded nanospheres is reported in Figure 2 and shows increasing total weight loss with increasing AL concentration.

**Figure 3:**
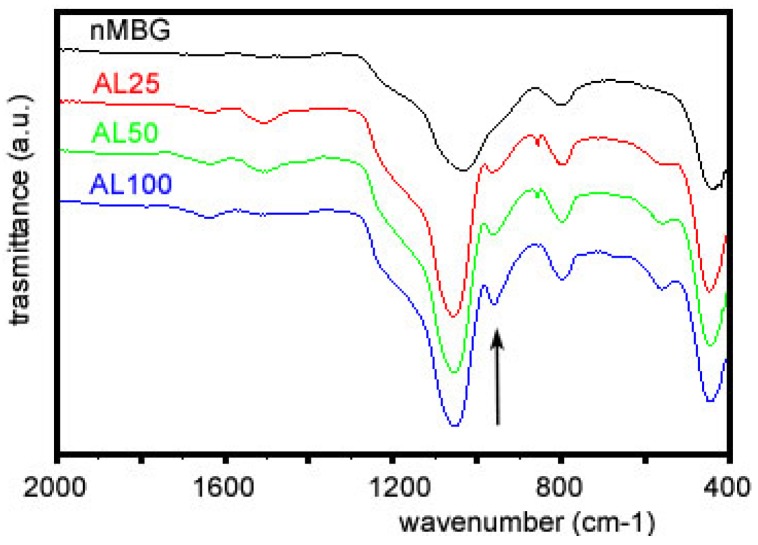
Fourier transform infrared spectroscopy (FT-IR) adsorption spectra of of the samples with different alendronate content. The band indicated by the arrow can be attributed to alendronate.

The presence of AL can be also detected by FTIR analysis (Figure 3) by the appearance of an absorption band around 960 cm^−1^, which can be attributed to the P–O bending vibrations of the AL structure 1088–920 cm^−1^, normally observed in the 1120–950 cm^−1^ range for organo-phosphorous compounds [37].

TEM images show that the morphology of the nanospheres is not significantly modified by AL loading (Figure 1d). However, AL incorporation provokes a significant decrease of the surface area and of the mean pore volume on increasing the bisphosphonate content up to about 17% (Table 1), suggesting a preferential interaction of the bisphosphonate with calcium ions on the pore surface.

**Figure 4:**
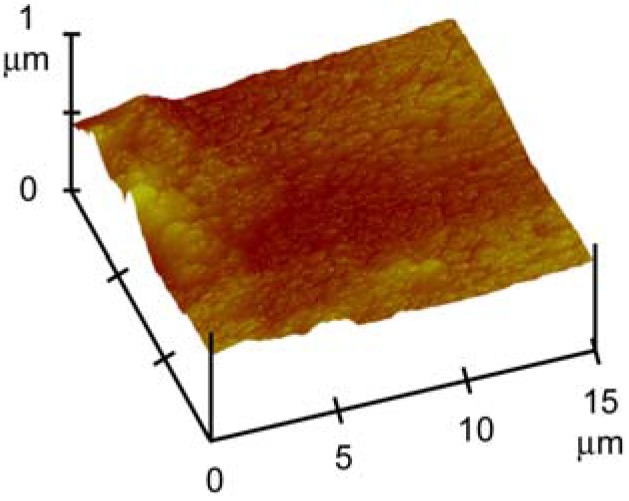
Atomic force microscopy (AFM) image of the surface of the nMBG disk-shaped sample.

*In vitro* mineralization, as well *in vitro* tests with osteoblast-like cells, were performed on disk shaped samples, which were obtained by pressing the powders into cylindrical molds. The surfaces of the disk-shaped samples are quite smooth, although they allow the nanospheres to be distinguished, as shown in the AFM image reported in Figure 4. The roughness parameters, *R*_a_, *R*_q_, and *R*_max_, do not vary significantly with composition and exhibit mean values of *R*_a_ = 53.60 ± 3 nm, *R*_q_ = 80.56 ± 9 nm, and *R*_max_ = 533.08 ± 70 nm. Accordingly, the values of contact angles are about 20° and do not vary as a function of AL content, thus indicating that all the samples display hydrophilic behavior. Slight differences can be appreciated in the time taken by the water droplet to completely spread on the sample surface, which range from 0.2 s for nMBG to 0.6 s for AL50 and to 1 s for AL100. This could be justified by the decreasing pore volume in these materials.

**Figure 5:**
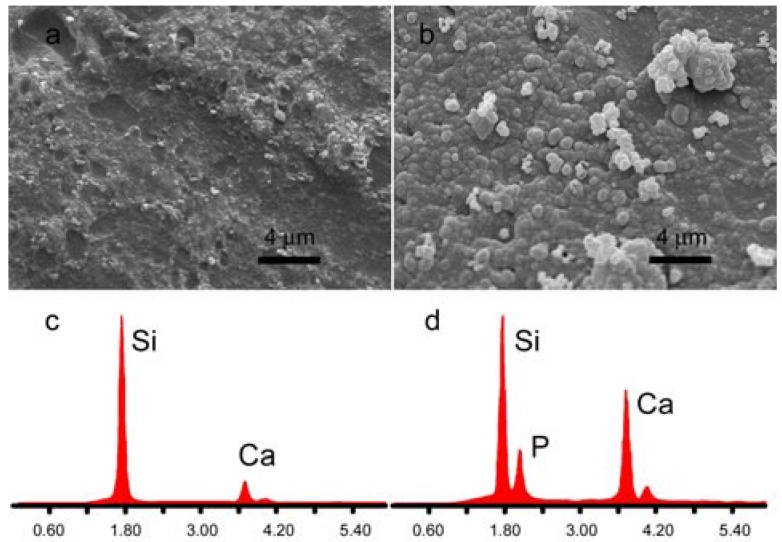
Scanning electron microscopy (SEM) images of disk-shaped samples surface as-prepared (**a**) and after immersion in simulated body fluid solution (SBF) for 1 week (**b**); Energy dispersive X-ray analyses (EDX) of disk-shaped sample surface as-prepared (**c**) and after immersion in SBF for 1 week (**d**).

Thanks to the presence of calcium, both nMBG and AL-loaded nanospheres promote mineralization from SBF. SEM images of the samples after immersion in SBF for one week show that the surface of the different disks are completely covered with apatite spheroids [30], which exhibit a mean diameter of about 500 nm (Figure 5).

Energy dispersive X-ray analysis (EDX) shows that nMBG exhibit only Ca and Si signals, whereas a distinct P signal can be detected after immersion in SBF. (Figure 5). No significant difference, in terms of morphology and composition, was appreciated between the deposits laid down on nMBG and AL-loaded nanospheres.

### 3.2. *In vitro* Cell Analysis

**Figure 6:**
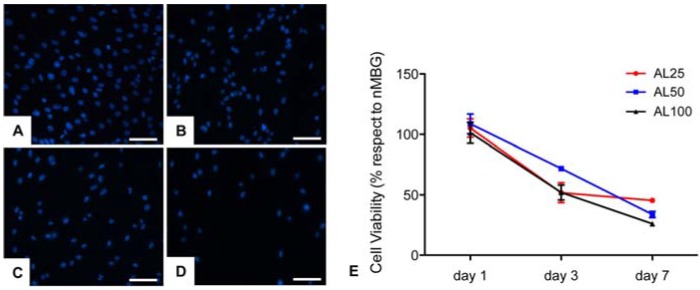
Fluorescence images of cell nuclei of MG63 cells seeded on the samples: (**A**) nMBG; (**B**) AL25; (**C**) AL50; and (**D**) AL100. Scale bars 100 µm. Cell viability (% with respect to nMBG) till day 7 (**E**).

The first part of the biological study was focused on the investigation of the proposed systems as potential inhibitors of osteosarcoma cell viability. MG63 cells were directly seeded on the upper surface of the samples and, as shown in Figure 6A–D, a consistent difference in cell density was seen starting from day 3 between the nMBG group and the others groups. A further demonstration of this cell viability reduction was seen with the MTT test (Figure 6E). It is remarkable that substantial decrease in cell proliferation (≈50% with respect to the nMBG group) was detected at day 3 and day 7 without any statistical differences among the group. These results proved the direct effect of our system on decreasing tumor cell viability even at the lower concentration of alendronate, in agreement with the suggested direct role of BPs in cancer treatment [38,39]. Even if the mechanisms of anticancer action of BPs are not completely known, it has been shown that BPs can exert direct cytostatic and antiproliferative effects against a variety of tumor cells including osteosarcoma, and metastasis in some tumors such as breast cancer, renal cell carcinoma, and prostate cancer [40,41,42].

**Figure 7:**
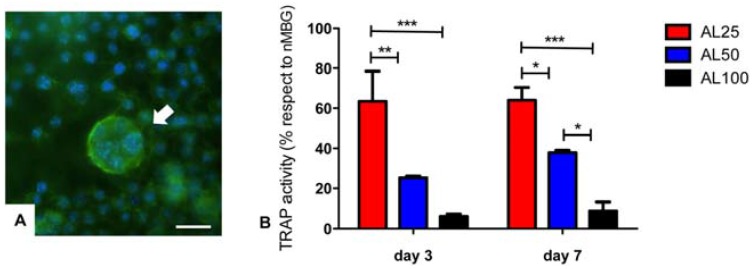
(**A**) sRANKL-treated RAW 264.7 cells of nMBG, arrow indicates a multinucleated osteoclast (OCLs) (actin in green, cell nuclei in blue). Scale bar 50 µm; (**B**) Tartrate resistant acid phosphatase (TRAP) activity shown as % with respect to nMBG used as control. * *p* ≤ 0.05; ** *p* ≤ 0.01; *** *p* ≤ 0.001.

A further aspect of the proposed systems is related to the inhibition of OCLs’ activity. Due to the fact that the aggressiveness of OS is associated with an increase of OCLs’ activity, osteoclast-targeted therapy is potentially an important avenue for addressing the progression of local and systemic OS disease. The RAW 264.7 cells were seeded in 24-well inserts, in the presence of sRANKL to induce osteoclastogenesis, and the samples with different amounts of alendronate were placed one per well in the 24-well plate. The presence of large and multinucleated cells showing a typical apico-basal actin-rich structure in nMBG group indicate that cells were grown regularly during the experiments and confirm the OCLs’ differentiation model (Figure 7) [43,44]. TRAP is physiologically highly expressed by active OCLs, and the results of this study show a strong, alendronate dose-dependent, reduction of TRAP activity (Figure 7B). In detail, AL100 shows the strongest TRAP activity reduction with respect to the nMBG group (TRAP activity < 10%) at both time points with statistical differences *versus* AL25 (*p* < 0.001 at day 3 and day 7) and *versus* AL50 (*p* < 0.5 at day 7). Furthermore, TRAP activity of AL50 results significantly reduced with respect to the nMBG group (TRAP activity < 40%), showing statistical differences with AL25 at both time points (*p* < 0.01 at day 3 and *p* < 0.05 at day 7). It is remarkable that even with the lowest amount of alendronate, AL25, there is a notable TRAP activity reduction proving that the proposed systems are promising drug delivery biomaterials.

**Figure 8:**
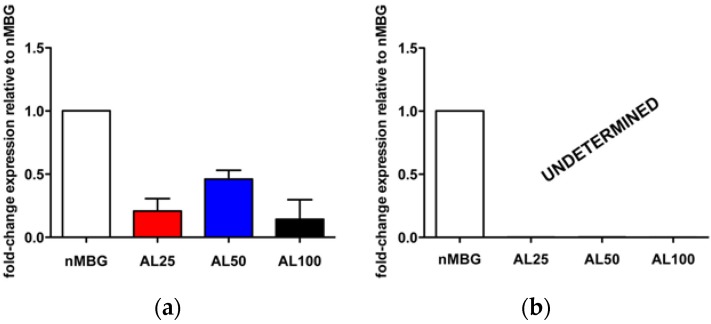
OCLs gene expression analysis. Relative quantification (2^−ΔΔ*C*^_t_) of gene expression after 7 days of OCLs cultured in direct contact with all the tested samples. Average and standard error of the technical triplicate of CtsK (**a**) and Oscar (**b**), with respect to the expression of the cells of nMBG, are indicated.

Moreover, in order to confirm the strong OCLs’ inhibition due to the presence of functionalized biomaterials, mRNA levels of Oscar and CtsK, typical osteoclastogenesis markers, were measured. In detail, Oscar is an OCLs’ associated receptor, its expression follows TRAP during osteoclasts differentiation [45]; CtsK is highly and quite selectively expressed in osteoclasts and it is a key protease in degradation of bone matrix molecules [46]. The results show a down-regulation of CtsK in the groups with alendronate compared to the nMBG group used as control without statistical differences among the AL groups (Figure 8). Interestingly, Oscar mRNA expression is completely inhibited by the presence of alendronate, in factthe mRNA level is undetectable (Figure 8).

## 4. Conclusions

The approach developed in this study allows mesoporous bioglass nanospheres to be loaded with increasing amounts of alendronate up to about 17 wt % and yields materials where the ability to promote mineralization of the nanospheres is coupled with the antiresorptive and anticancer properties of alendronate. The results of *in vitro* tests prove that the proposed multifunctional system is a promising platform for controlled regulation of osteosarcoma cells and osteoclast activity, providing a new tool for OS treatment.

## Figures and Tables

**Table 1:** Specific surface areas, pore volumes, and alendronate (AL) content of samples obtained on increasing concentrations of AL in solution.

Sample	Specific Surface Area (m^2^/g)	Pore Volume (cm^3^/g)	AL Content (wt %)
nMBG	528.4 ± 0.9	0.63 ± 0.02	-
AL25	327.3 ± 0.3	0.65 ± 0.02	4.7 ± 0.2
AL50	349.5 ± 0.4	0.36 ± 0.01	9.0 ± 0.4
AL100	165.3 ± 0.3	0.44 ± 0.01	17.0 ± 0.9
